# Nitrogen-Doped Carbon-Coating Disproportionated SiO Materials as Long Cycling Stable Anode for Lithium Ion Batteries

**DOI:** 10.3390/molecules26061536

**Published:** 2021-03-11

**Authors:** Ben Huang, Binbin Chu, Tao Huang, Aishui Yu

**Affiliations:** 1 Collaborative Innovation Center of Chemistry for Energy Materials, Shanghai Key Laboratory of Molecular Catalysis and Innovative Materials, Department of Chemistry, Institute of New Energy, Fudan University, Shanghai 200438, China; bhuang18@fudan.edu.cn (B.H.); 18110220027@fudan.edu.cn (B.C.); 2 Laboratory of Advanced Materials, Fudan University, Shanghai 200438, China

**Keywords:** lithium ion batteries, anode, silicon, carbon coating, nitrogen-doped, high energy density

## Abstract

Silicon monoxide (SiO) is a kind of promising anode material for lithium-ion batteries because of its smaller volume change during the charge and discharge process than pure silicon and its higher theoretical capacity than commercialized graphite. However, its fast-fading capacity still restricts the development of practical application of SiO. A simple and cheap strategy to dope nitrogen and coat carbon on the surface of disproportionated SiO is proposed to improve the cycling stability significantly even at a high specific current. The capacity retention is nearly 85% after 250 cycles and more than 69% after 500 cycles at a specific current of 1000 mA g^−1^. Even at a specific current of 2000 mA g^−1^, its cycling performance behaves similarly to that of 1000 mA g^−1^. Nitrogen doping in materials could improve the conductivity of materials because pyridinic nitrogen and pyrrolic nitrogen could improve the electron conductivity and provide defects to contribute to the diffusion of lithium ions. The use of pitch and melamine, which are easily available industrial raw materials, makes it possible to contribute to the practical application.

## 1. Introduction

Nowadays, lithium ion batteries (LIBs) have conquered the portable electronics and new-energy vehicles markets of because of their advantages in energy density, lifespan and positive relationship to the environment. LIBs have become known as one of the most attractive energy storage devices, providing significant contributions in modern society. Meanwhile, the fast development of new energy vehicles and portable electronics has also stimulated the development and investigation of LIBs with higher energy and power density. In 2019, three scientists won the Nobel Prize in Chemistry for their excellent contributions to the development of LIBs [1]. A growing number of studies have been investigated for LIBs with higher energy density and higher cycling stability [2,3].

The energy density of LIBs mainly depends on their cathode and anode materials. For cathode materials, many kinds of materials, such as LiCoO_2_, LiNi_x_Co_y_Al_1−x−y_O_2_, LiNi_x_Mn_y_Co_1−x−y_O_2_, etc., were investigated and modified to improve their capacity, cycling performance, rate performance and other capabilities [4,5,6], while their actual specific capacity is close to their theoretical capacity, which means that it is difficult to improve their capacity significantly. For anode, carbon-based materials were widely used in commercialized batteries because they perform well in cycling and rate performance. However, to improve the energy density further, new materials must be investigated because the theoretical specific capacity of carbon (372 mA h g^−1^, LiC_6_) is relatively low [7]. Silicon (Si)-based materials were found to display the highest theoretical specific capacity (4200 mA h g^−1^ for Li_22_Si_5_ at 400 °C, generally is 3579 mA h g^−1^ for Li_15_Si_4_ at room temperature) among all anode materials for LIBs [8,9,10,11,12,13,14,15]. However, pure silicon undergoes drastic volume changes during the charging and discharging process, leading to an unstable Si structure and solid electrolyte interphase (SEI) layer, causing a poor cycling lifespan [11,16,17]. Silicon monoxide (SiO) delivers a lower volume change than pure silicon. It is also abundant, cheap and could be produced easily, which means that SiO is a possible anode material that can replace pure Si for next generation lithium ion batteries [18]. However, SiO also suffers from several serious problems such as low intrinsic electrical conductivity, non-negligible volume change and low initial Coulombic efficiency (ICE) [18]. For ICE, some methods of pre-lithiation on anode materials or electrodes have been reported. Our group has also reported a kind of pre-lithiation method using stabilized lithium metal powder (SLMP) to improve ICE easily [19]. However, it could not improve the cycling performance. SiO material must be modified before it could be used as anode material for lithium ion batteries in practical application.

For bare SiO, many strategies, such as downsizing, constructing porous structures, nano-compositing were used to solve the problem of drastic volume change [20,21,22,23,24,25]. Sohn et al. employed high-energy mechanical milling (HEMM) process to combine disproportionated SiO (d-SiO) based anode combined with nanosized Si. This kind of material delivered a high reversible specific capacity of about 1000 mA h g^−1^ with good capacity retention [22]. NaOH solution was used to etch SiO_2_ matrix in d-SiO to produce porous SiO_x_ without carbon coating, which demonstrated a high specific capacity of around 1240 mA h g^−1^ even after 100 cycles [20]. These strategies make bare SiO materials behave a relatively stable cycling performance with a high specific capacity. However, most obtained specific capacities were based on a low current density. For SiO/C composite, it is easy to deliver a better cycling performance because of the excellent electrochemical performance of C. A kind of SiO/C composite prepared by Ohzuku’s group manifested a discharge capacity of 700 mA h g^−1^ after 100 cycles [26]. Oh et al. also reported a micrometer-sized d-SiO/C composite with good electrochemical performance [27,28]. Though the compositing with C is a good way to improve cycling stability, the prepared active materials perform poor rate capability. To tackle this issue, N element was used to dope in the coating layer. Tu et al. proposed a kind of anode material with core-shell structure of porous silicon and N-doped C layer (p-Si/NC) [29]. There were three kinds of N atoms in the coating layer, leading to produce active sites in C layers which could contribute the lithium ions diffusion. This kind of electrode delivered a capacity of 750 mA h g^−1^ after 200 cycles under 200 mA g^−1^ with a capacity retention of 73%. It also performs well in rate performance. Choi et al. fabricated N-doped carbon coated SiO (SiO/NC) using N-containing ionic liquid, delivering enhanced capacity and rate capability [30]. However, this strategy is not suitable in industry because of the high cost of ionic liquid. Cheap raw materials need to be searched and investigated to make N-doped C-coating SiO based materials available to the production of industry.

In this work, cheap and available pitch and melamine were used as C source and N source to coat disproportionated SiO (d-SiO). The prepared active material (d-SiO-NC) delivered relative stable cycling stability especially at a high current density. This strategy is easy to be operated in industrial production because of the convenience of the process and the low price and availability of raw materials. The capacity retention of this material arrived 85% after 250 cycles and 69% after 500 cycles at a specific current of 1000 mA g^−1^. Besides, there is no obvious change in reversible specific capacity between the specific current of 1000 and 2000 mA g^−1^. Good cycling capability and rate performance make it become a promising anode material in next generation lithium ion batteries.

## 2. Results and Discussion

### 2.1. Materials Preparation

**Figure 1:**
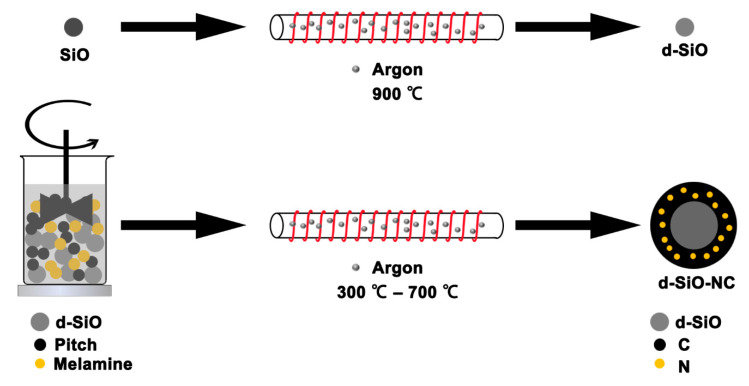
Schematic illustration of preparing nitrogen-doped carbon-coating disproportionated silicon monoxide (d-SiO-NC).

Figure 1 shows the schematic illustration of preparing N-doped C-coating disproportionated SiO (d-SiO-NC). When pristine SiO was heated to 900 °C under an argon atmosphere, SiO was partly disproportionated. [31] According to the literature report, there would be three different kinds of N in the coating layer after coating process [29], which would produce some defects in the C layer and further contribute to the lithium ions transport. To sum up, the disproportion of SiO, N-doped and C-coating improve the electrochemical performance jointly.

### 2.2. Materials Morphology

**Figure 2:**
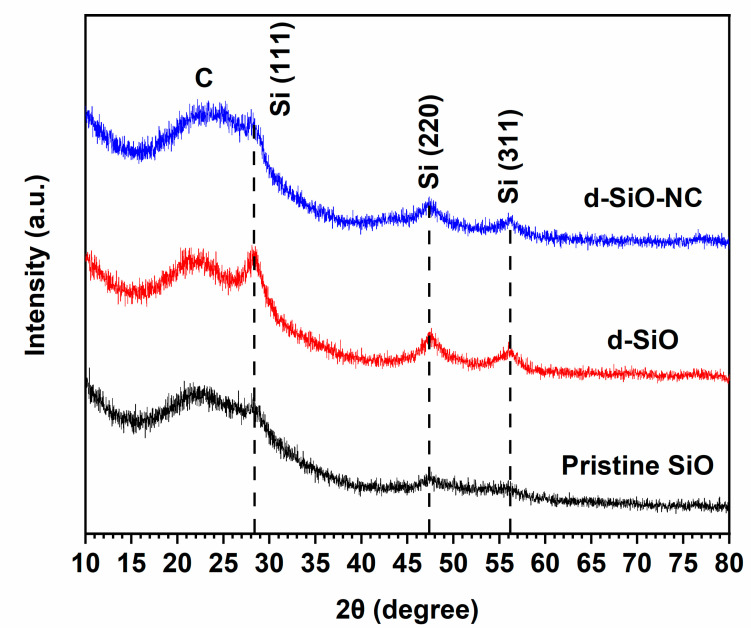
XRD patterns of pristine SiO, d-SiO, and d-SiO-NC (2:1.5:3).

XRD patterns were used to characterize pristine SiO, d-SiO, and d-SiO-NC (2:1.5:3) materials (Figure 2). Compared with pattern of pristine SiO and d-SiO, the diffraction peak at about 28.4°, 47.4° and 56.2° corresponding to the typical plane of Si (111), Si (220) and Si (311) [32] are stronger in pattern of d-SiO than those of SiO, indicating that after heated at 900 °C, crystal Si was produced successfully after disproportion of SiO. Besides, a broad peak at about 23.4° appeared in the pattern of d-SiO-NC, which suggests that carbon was obtained during the carbonization process.

**Figure 3:**
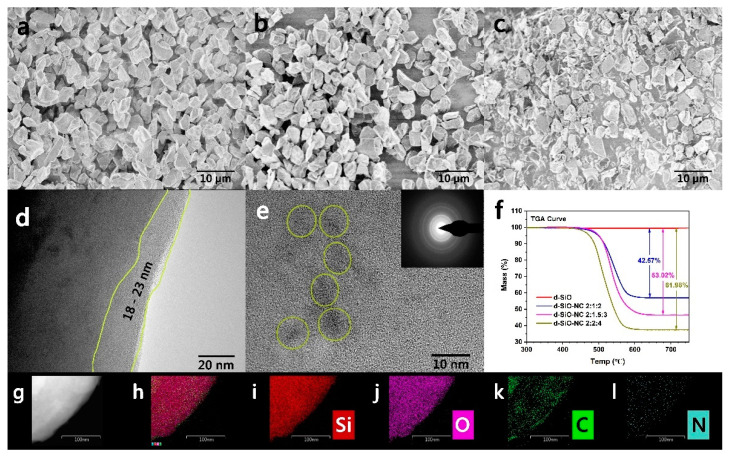
SEM patterns of pure SiO (**a**), d-SiO (**b**) and d-SiO-NC (2:1.5:3) (**c**). (**d**) TEM images of the coating layer of d-SiO-NC (2:1.5:3). (**e**) TEM images of the inner part of d-SiO-NC (2:1.5:3) with the corresponding lattice spacing. (**f**) TG curve of d-SiO-NC (2:1.5:3) with different ratio of raw materials. (**g**–**l**) Element mapping images of d-SiO-NC (2:1.5:3) (Scale bar: 100 nm).

The morphology of materials was characterized and investigated by SEM and TEM (Figure 3a–e). Pristine SiO is combined with a large amount of small and uniform particles with an average diameter of 3–6 μm (Figure 3a). After heated, there is no obvious change in the particles’ shape and size (Figure 3b). Different sizes of particles could be found in the SEM pattern of d-SiO-NC (Figure 3c), suggesting that N and C may coat on the surface of d-SiO successfully. In the TEM pattern of d-SiO-NC (Figure 3d), a layer with about 20 nm thick could be found on the surface of particles, further proving that d-SiO was coated successfully. Some lattice fringe of Si could be found in the HRTEM pattern and selected-area diffraction (SAD) of d-SiO-NC (Figure 3e), indicating that some crystal Si was produced during the disproportion process. The formation of crystal Si would contribute to improving specific capacity, while partially developed several-nanometers Si performs a poor volume change. According to the TEM element mapping image (Figure 3g–l), N was uniformly doped in the active materials, while C was also distributed in the particles.

Different mass ratios of d-SiO, melamine and pitch (2:2:4, 2:1.5:3 and 2:1:2) were used to prepare d-SiO-NC materials. To confirm the element content, thermogravimetric analysis (TGA) was operated on three different kinds of synthesized materials. TGA curve was shown in Figure 3f. The content of d-SiO could be calculated according to the curve. After calculation, the mass ratio of d-SiO in three different kinds of materials are 57.43%, 46.88% and 38.02%. After further calculation, the contents of N and C in three materials could be calculated and shown in Table 1.

**Figure 4:**
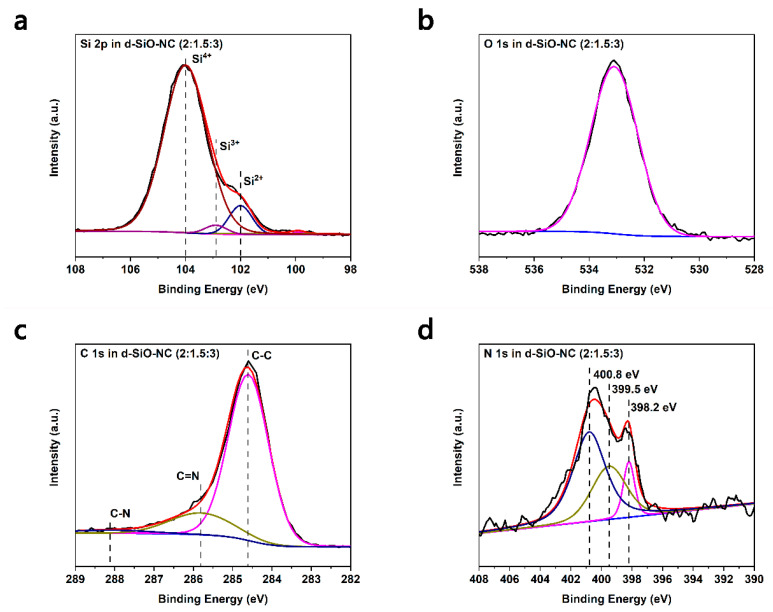
XPS spectra of d-SiO-NC (2:1.5:3). (**a**) Si 2p, (**b**) C 1s, (**c**) O 1s, (**d**) N 1s.

To further confirm the chemical state on the particle surface of the composites, XPS experiments were performed. The binding energies were calibrated with respect to the C 1s peak at 284.6 eV. Figure 4a shows the high-resolution spectrum of Si 2p. The peaks at 104 eV, 102.9 eV and 102 eV represent Si^4+^, Si^3+^ and Si^2+^, respectively, indicating that there is a large amount of SiO_2_ on the surface of particles, while the peak at 99.9 eV, which represents Si^0^, is barely visible. The disproportion reaction might lead to produce more crystal Si in the particle while more SiO_2_ on the surface of the particle [33,34]. Figure 4b displays the spectrum of O 1s. There is one main peak (533.1 eV) corresponding to the Si-O bond that could be found. Figure 4c shows the spectrum of C 1s. The peak can be divided into three peaks at 284.6 eV, 285.8 eV and 288.1 eV, which represent C-C, C=N and C-N bonds, respectively [29,35]. The appearance of peaks corresponding to the C=N and C-N bond suggests that N has been successfully doped into the layer of carbon. It could be also indicated that double bonds exist more between C and N. Figure 4d shows the spectrum of N 1s. Three peaks referring to pyridinic N (398.2 eV), pyrrolic N (399.5 eV) and quaternary N (400.8 eV) could be divided after split process [29]. Among all three kinds of N, pyridinic N and pyrrolic N could improve the electron conductivity and provide defects to contribute to the diffusion of lithium ions.

### 2.3. Electrochemical Performance in Half Cells

**Figure 5:**
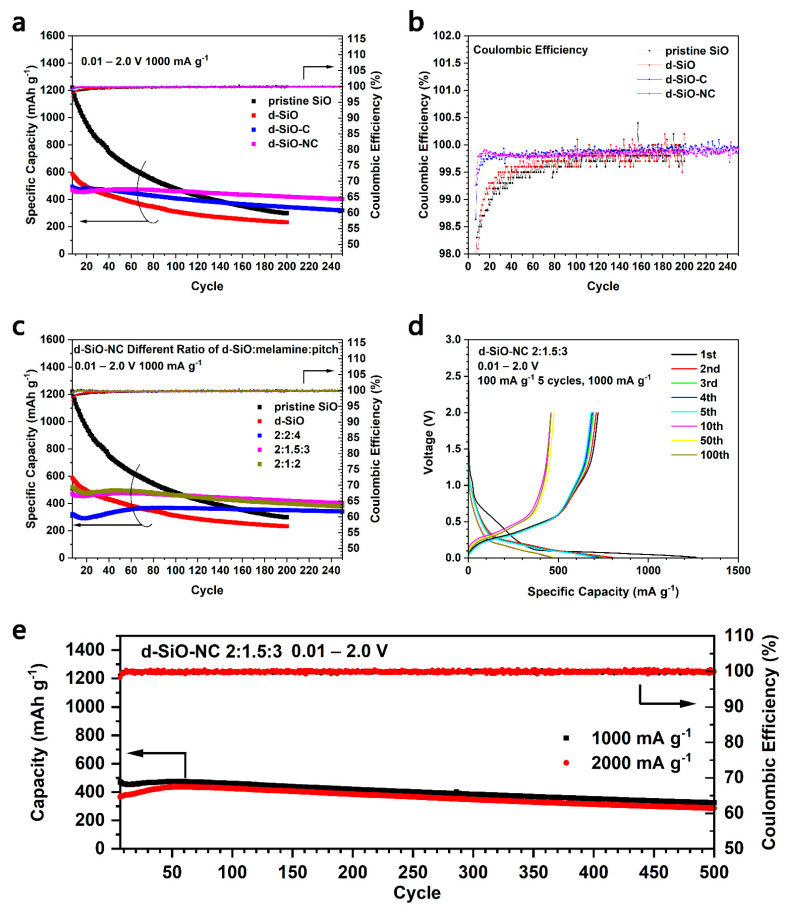
Cycling performance of half cells fabricated by pristine SiO, d-SiO, d-SiO-C (2:4.5) and d-SiO-NC (2:1.5:3) (**a**) and their Coulombic efficiency (**b**). (**c**) Cycling performance of half cells fabricated by pristine SiO, d-SiO and d-SiO-NC with different ratio of raw materials. (**d**) Charge/discharge voltage profiles of d-SiO-NC (2:1.5:3) in different cycles. (**e**) Long cycling performance at different current density of d-SiO-NC (2:1.5:3).

The cycling performances at a specific current of 1000 mA g^−1^ of pristine SiO, d-SiO, d-SiO-C (2:4.5) and d-SiO-NC (2:1.5:3) are shown and compared in Figure 5a. The specific capacity of SiO and d-SiO show an obvious decrease as the cycling process, while after coating, the capacity retention improved a lot. Compared to pure C-coating (blue squares) with N-doped C-coating (pink squares), N-doped C-coating d-SiO performs a more stable cycling performance. After 250 cycles at a specific current of 1000 mA g^−1^, its specific capacity was still more than 400 mA h g^−1^ with a capacity retention of more than 85%, showing a more stable cycling performance than that of only C-coating. Figure 5b shows the detailed Coulombic efficiency of pristine SiO, d-SiO, d-SiO-C (2:4.5) and d-SiO-NC (2:1.5:3). It could be indicated that the Coulombic efficiency improves quickly to nearly 99.8% and keeps stable after coating. d-SiO-NC (2:1.5:3) performs best among all four kinds of materials considering Figure 5a,b. On the other hand, considering the effect of different ratios of raw materials, active materials composed with different ratios of d-SiO, melamine and pitch (2:2:4, 2:1.5:3 and 2:1:2) were prepared to investigate their cycling performance. Figure 5c shows the comparison of cycling performance among them. When the mass ratio of d-SiO, melamine and pitch is 2:2:4, d-SiO-NC shows a relatively stable cycling performance; however, its reversible specific capacity is really low. d-SiO-NC (2:1.5:3) and d-SiO-NC (2:1:2) both perform good cycling stability and high specific capacity, in which d-SiO-NC (2:1.5:3) shows higher capacity retention (85%) than d-SiO-NC (2:1:2) (73%) after 250 cycles at a specific current of 1000 mA g^−1^. Figure 5d shows the charge/discharge voltage profiles of d-SiO-NC (2:1.5:3) at different cycles. Its first discharge voltage profile shows a distinct plateau at 1.1 V, which represents the formation of the SEI layer. There were no obvious changes among the charge/discharge voltage profile at 10, 50 and 100 cycles, indicating the relative stability of this kind of material. To verify its cycling stability further, d-SiO-NC-based half cells were cycled for 500 cycles at two different specific currents of 1000 and 2000 mA g^−1^. The obvious change of specific capacity between two currents could be only found before the 50th cycle, while the specific capacity increased obviously at 2000 mA g^−1^ because enough time and cycles are necessary for materials activation and sufficient infiltration of electrolyte. After 50 cycles, the half cells both show a stable performance and high capacity retention (69% and 66% at 1000 and 2000 mA g^−1^, respectively). Besides, the difference of specific capacities at two different currents is also relatively small, indicating that this kind of anode material could also be applied in some situations under a high current density.

**Figure 6:**
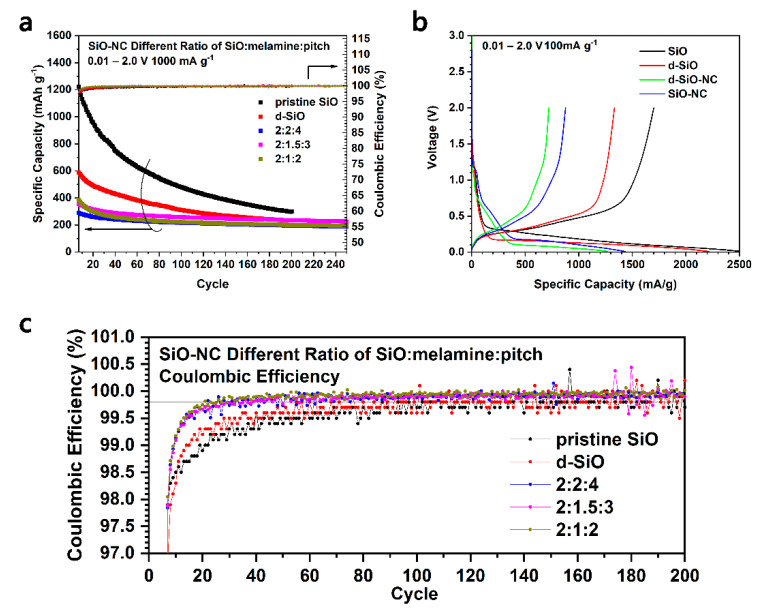
Cycling performance (**a**) and Coulombic efficiency (**c**) of pristine SiO, d-SiO, SiO-NC with different ratio of raw materials. (**b**) First charge/discharge voltage profile of SiO, d-SiO, d-SiO-NC (2:1.5:3) and SiO-NC (2:1.5:3).

In Figure 5a,c, it could be found that when SiO was heated to produce d-SiO, their cycling performance decreased significantly. It seems that a higher specific capacity can arrive if pristine SiO was coated with C directly. To verify this idea, SiO-NC was prepared and used to fabricate half cells for the cycling performance test (Figure 6). However, SiO-NC only shows a relatively low cycling capacity in all mass ratios of SiO, melamine and pitch (blue, pink and dark yellow squares in Figure 6a). Meanwhile, SiO-NC also delivers a larger irreversible capacity during the first discharge process than d-SiO-NC (Figure 6b). In Figure 6c, it could be indicated that SiO-NC-based half cells would get a Coulombic efficiency of 99.8% after about 20 cycles, which is slower than d-SiO-NC-based half cells (about 10 cycles) (Figure 5b). All mentioned above demonstrated that d-SiO-NC performs well than SiO-NC as anode for lithium ion batteries. This may because that crystal Si produced after heated may help provide a higher capacity; however, the disadvantage of pure Si would perform significantly if there is not a coating layer. Compared with the results reported in other literatures [29,30,36,37], d-SiO-NC also performs a better capability. Though similar capacity and cycling performance could be obtained in some of them, the specific current was usually relatively low, or expensive raw materials and complicated method was used during their synthesis process.

### 2.4. CV and EIS Test and the Calculation of Chemical Diffusion Coefficient

**Figure 7:**
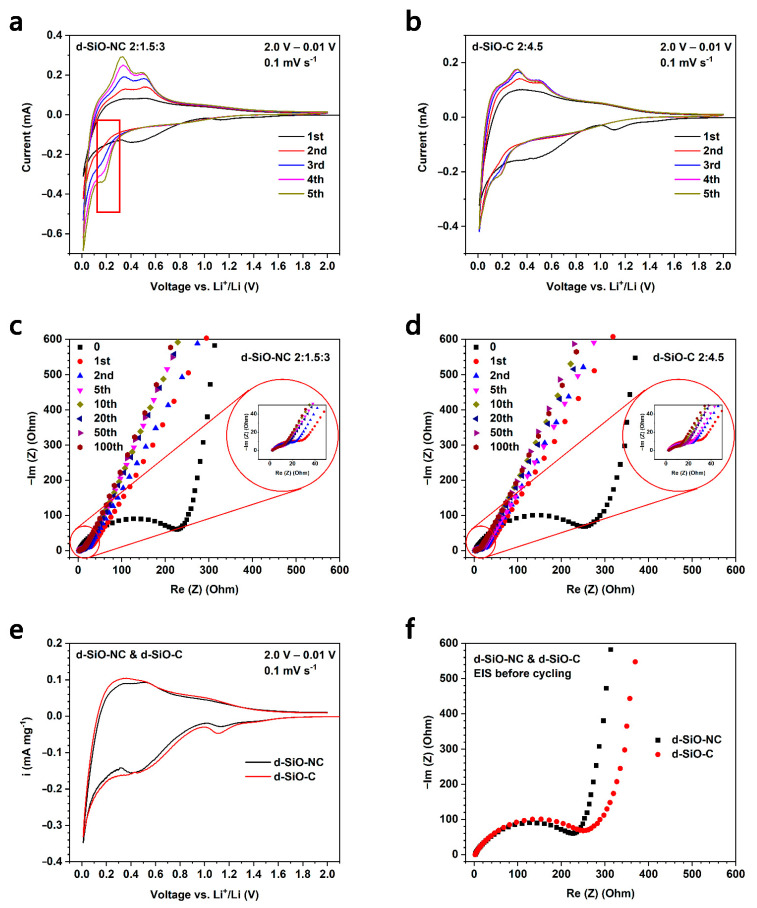
Cyclic voltammetry curve of first 5 cycles of d-SiO-NC (2:1.5:3) (**a**) and d-SiO-C (2:4.5) (**b**). Electrochemical impedance spectroscopy after different cycles of d-SiO-NC (2:1.5:3) (**c**) and d-SiO-C (2:4.5) (**d**). Comparison of first-cycle CV curve (**e**) and EIS spectroscopy (**f**) between d-SiO-NC (2:1.5:3) and d-SiO-C (2:4.5). (**g**) The equivalent circuits of EIS analysis.

To investigate the difference between N-doped and non-N-doped, CV and EIS tests were operated on the lithium half cells based on d-SiO-NC (2:1.5:3) and d-SiO-C (2:4.5) materials. Figure 7a,b show the CV curve of the first five cycles of d-SiO-NC (2:1.5:3)-based half cells and d-SiO-C (2:4.5)-based half cells. It can be found that during the first anode scan, a small peak at around 1.1 V and a broad peak at around 0.4–0.5 V can be found in both two graphs, while during the second anode scan, these two peaks disappeared, indicating that these two peaks are related to the decomposition of FEC in electrolyte, the formation of SEI layer and other irreversible reaction during the first discharge process. The peak at 0.2–0.3 V during the anodic scan represents the lithiation of C and d-SiO materials. The peaks at 0.4 and 0.6 V during the cathodic scan represent the de-lithiation of d-SiO. The peak shape during the first cycle is significantly different from that of the following cycles, suggesting that the formation of the SEI layer and other irreversible reactions might consume a large amount of active lithium, causing a low initial Coulombic efficiency (ICE). This could also be demonstrated in cycling performance, which shows a low ICE of 56%. To improve the ICE, SLMP was used to pre-lithiate d-SiO-NC (2:1.5:3) electrode. More details about pre-lithiation are described in the next part. Compared with Figure 7a,b, the intensity of the peak of d-SiO-NC (2:1.5:3)-based half cells increases faster than that of SiO-NC (2:4.5)-based half cells, indicating that the doping of N could accelerate the activation of active materials. This property can be used to reduce the formation time of batteries. Compared with the first cycle CV curve of two kinds of materials (Figure 7e), the peak at around 1.1 V of d-SiO-NC delivered lower intensity than that of d-SiO-C, while at around 0.4–0.5 V, the peak of d-SiO-NC is more obvious than that of d-SiO-C, indicating that the mechanism of active lithium loss during the first discharge process of two kinds of materials is different. N may restrict the irreversible reaction at 1.1 V, while it contributes it at 0.4–0.5 V.

In Figure 7c,d, the EIS spectra after different cycles of both d-SiO-NC (2:1.5:3) and d-SiO-C (2:4.5) based half cells are shown. “0” means the EIS was operated before starting cycling. The comparison of EIS spectra during the first cycle between two materials is shown in Figure 7f. These EIS spectra were fitted based on the equivalent circuit shown in Figure 7g and the fitting results are shown in Table 2. *R_s_* represents the ohmic resistance (resistance between the electrode and electrolyte), *R_f_* means the resistance of solid electrolyte film and *R_ct_* represents the resistance of charge transfer. The inclined line is corresponded to the lithium ion diffusion process, representing the Warburg impedance (*Z_w_*). According to the fitting results in Table 2, it suggested that the *R_s_* is similar in two kinds of cells, while the *R_f_* and *R_ct_* of d-SiO-NC based cells are lower than d-SiO-C based cells. Before cycling, the *R_f_* reduced from 285.3 Ω to 243.2 Ω after N-doping. the results after cycling also shows an obvious decrease. In conclusion, these EIS spectra and fitting results suggests that d-SiO-NC (2:1.5:3) could provide better lithium ion transfer conductivity. These results are in accordance with the results of the long cycling performance at a high current density.

To investigate the transport of lithium ions further, the lithium ion chemical diffusion coefficient (*D_Li^+^_*) could be calculated by EIS and CV method according to the literature [38]. Using EIS method, *D_Li^+^_* can be calculated by the following equation:

(1) $$D_{Li^{+}}=\frac{R^{2}T^{2}}{2n_{Li^{+}}^{4}A^{2}F^{4}C_{Li^{+}}^{2}\sigma_{W}^{2}}$$

**Figure 8:**
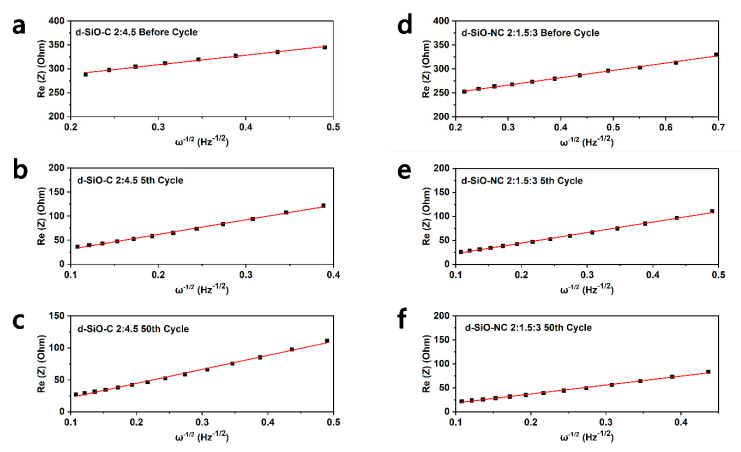
The relationship between Re(Z) and square root of inverse angular frequency, *ω*^−1/2^ in low frequency region for d-SiO-C//Li cell (**a**) before cycle, (**b**) after 5th cycle and (**c**) after 50th cycle, and d-SiO-NC//Li cell (**d**) before cycle, (**e**) after 5th cycle and (**f**) after 50th cycle.

In this equation, *R* represents the gas constant (8.314 J mol^−1^ K^−1^), *T* represents temperature in Kelvin (298.15 K), *n_Li^+^_* represents the number of electrons per species reaction (for lithium ions, *n_Li^+^_* = 1), A represents the surface area of electrode (1.131 cm^2^), *F* means Faraday constant (96485 C mol^−1^), *C_Li^+^_* represents the concentration of lithium ions in mol cm^−3^ (0.06323 mol cm^−3^ and 0.05822 mol cm^−3^ for d-SiO-C (2:4.5) and d-SiO-NC (2:1.5:3) respectively calculated by molar mass and density of two kinds of active materials) and *σ_W_* means the Warburg coefficient, which can be obtained in the real part of impedance (Re(Z)) vs. *ω*^−1/2^ graphs (Figure 8). The relationship between Re(Z) and square root of inverse angular frequency (*ω*^−1/2^) can be described as equations in Equation (2) and the slope represents the *σ_W_*.

(2) Re Z=Rohmic+Rct+σWω−1/2

In this equation, *ω* represents the angular frequency, which is the same as *2πf* in which f means the frequency in Hertz. After linear fitting of Re(Z) and *ω*^−1/2^, *σ_W_* can be calculated in after different cycles of cells, which is shown in Table 3. Based on equal (1), *D_Li^+^_* can be calculated and shown in Table 3. Compared the value in table, it could be indicated that the *D_Li^+^_* is higher in d-SiO-NC than d-SiO-C, demonstrating that the doping of N could contribute the transport of lithium ions.

**Figure 9:**
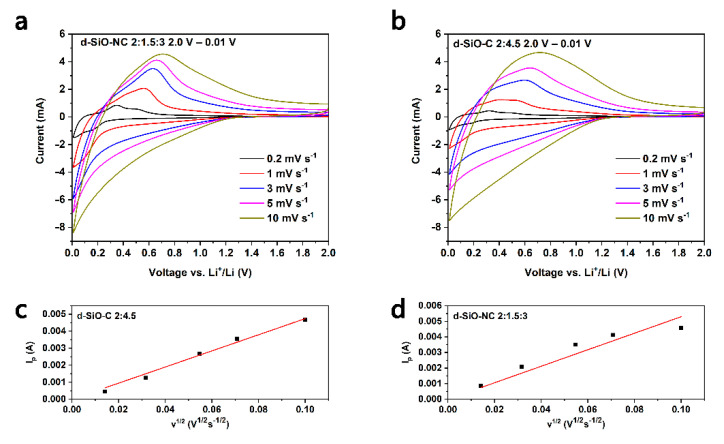
Cyclic voltammetry curves of d-SiO-C (2:4.5) (**a**) and d-SiO-NC (2:1.5:3) (**b**) in different scan rate. Peak current, *I_p_* as a function of square root of scan rate, *v*^1/2^ of d-SiO-C (2:4.5) (**c**) and d-SiO-NC (2:1.5:3) (**d**).

Figure 9a,b shows the CV characteristics of d-SiO-C (2:4.5) and d-SiO-NC (2:1.5:3) under different scan rates. It can be found that with the increase of scan rate, the peak current (*I_p_*) increases gradually and the peak potential in cathode scan shifts to higher on in both two kinds of cells. In CV curve under a scan rate of 0.1 mV s^−1^ (Figure 7a,b), two oxidation peaks and two reduction peaks could be found clearly, while if scan rate rises to more than 1 mV s^−1^, only one pair of redox peaks could be found, which is in accordance with Teo’s work [36]. Based on the *I_p_* in Figure 9a,b, in which displays the relationship between *I_p_* and *v*^1/2^, it could be recognized that the lithiation/de-lithiation processes are diffusion-controlled, and *D_Li^+^_* based on CV data could be calculated using the following Randles-Sevcik equation [38]:

(3) $$I_{p}=\left(2.69\times 10^{5}\right)n_{Li^{+}}^{3/2}AD_{Li^{+}}^{1/2}C_{Li^{+}}v^{1/2}$$

In this equation, *I_p_* represents the peak current of CV curves in ampere, and *v*^1/2^ represents the scan rate in mV s^−1^. The meanings of *n_Li^+^_*, *A*, *D_Li^+^_*, and *C_Li^+^_* have been mentioned previously. The slope of fitted line in Figure 9c,d could be used to calculate the *D_Li^+^_*. The results are 6.07 × 10^−12^ cm^2^ s^−1^ and 8.94 × 10^−12^ cm^2^ s^−1^ for d-SiO-C (2:4.5) and d-SiO-NC (2:1.5:3) respectively. The *D_Li^+^_* calculated by CV curves could also suggest that the doping of N could improve the transport of lithium ions. The results of *D_Li^+^_* calculated by CV analysis is about several orders of magnitude higher than that calculated by EIS analysis, which may because of the system and internal error. This phenomenon is accordance with results reported in other literatures [36].

### 2.5. Pre-Lithiate d-SiO-NC Anode and Full Cell Test

**Figure 10:**
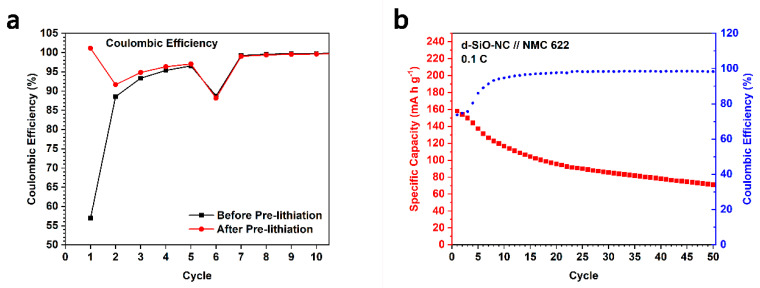
(**a**) Coulombic efficiency of first several cycles in cycling test of d-SiO-NC (2:1.5:3) before and after pre-lithiation. (**b**) Cycling performance of full cell fabricated by pre-lithiated d-SiO-NC (2:1.5:3) and LiNi_0.6_Mn_0.2_Co_0.2_O_2_ (NMC 622).

Though d-SiO-NC-based half cells deliver a stable cycling performance even at a high current, they perform a low ICE during the cycling process (approximately 56%), which is a fatal disadvantage in practical application. To enhance ICE, SLMP-SBR-Toluene (SST) suspension was used to pre-lithiate d-SiO-NC (2:1.5:3) anode. 16 μL SST suspension, with 1 wt% of SLMP, was added to the surface of the electrode. Then electrode with SST was treated in the same way as we reported before [19]. The initial Coulombic efficiency was improved to around 101% in half cells (Figure 10a). The Coulombic efficiency of the first several cycles is also higher than d-SiO-NC (2:1.5:3) without pre-lithiation. The pre-lithiated d-SiO-NC (2:1.5:3) electrodes were used to assemble full cells. The decrease of Coulombic efficiency at the 6th cycle is because the specific current was enhanced to 1000 mA g^−1^ (at the end of activation).

To verify its promise in practical application, LiNi_0.6_Mn_0.2_Co_0.2_O_2_ (NMC 622) was used as cathode material to assemble full cells. The electrolyte, separator in full cells and the process of assembling are the same as that of half cells. The assembled full cells were charged and discharged at a specific current of 0.1 C based on the mass of cathode materials. As shown in Figure 10b, after 40 cycles, the specific capacity of full cells could keep around 80 mA h g^−1^ (based on the mass of NMC 622). Meanwhile, there is also a stable Coulombic efficiency after around 10 cycles. This kind of material has a potential value to be used in lithium ion full cells.

## 3. Materials and Methods

### 3.1. Material Synthesis

All materials were obtained by producers and used in experiment without further treatment. In particularly, pitch, extracted from crude oil and mostly made up of C, is a kind of complex mixture composed with hydrocarbon in different molecular weight and other non-metal composites. The schematic illustration of the preparation of d-SiO-NC is shown in Figure 1. d-SiO was produced by the disproportion of SiO powder (Rijinwen Silicon Co., Peixian, China, 99.95%, D(50): 3 μm, specific area: 2.11 m^2^ g^−1^) at 900 °C under an argon atmosphere. d-SiO, pitch (Tianjin HX Chemicals Co., Ltd., Tianjin, China; softening point: 300 °C.) and melamine (AR grade, Sinopharm Chemical Reagent Co., Ltd., Shanghai, China) were mixed with a certain mass ratio in a beaker. 30–40 mL of DI H_2_O (deionized water) was added and the obtained suspension was dispersed under ultrasound for 20–30 min to make d-SiO, pitch and melamine dispersed evenly in H_2_O. Then the suspension was heated to 55–60 °C with mechanical stirring to solvent was evaporated completely. The residue was transferred to tube furnace and heated to 300 °C for 7 h in an argon atmosphere, then the temperature was increased to 700 °C for 3 h to make pitch and melamine carbonize. The d-SiO-NC was produced after cooling and used to prepare anode for lithium ion batteries directly. Three different ratios of d-SiO, melamine and pitch (2:2:4, 2:1.5:3 and 2:1:2) were prepared. For d-SiO-C and SiO-NC, the preparation methods are the same as mentioned above except that melamine was replaced by pitch or d-SiO was replaced by SiO.

### 3.2. Material Characterization

To analyze the morphology of active materials, XRD (D8 Discover, Bruker, karlsruhe, Germany) with Cu-Kα radiation was utilized. Field-emission scanning electron microscopy (FESEM, S-4800, Hitachi Co., Tokyo, Japan) was used. X-ray photoelectron spectroscopy (XPS, Perkin Elmer Co., Waltham, MA, USA) was adopted to analyze the N and C on active materials particles using Al Kα radiation (hν = 1486.6 eV). The microstructure of the coating layer and the distribution of elements were observed by transmission electron microscopy (TEM, JEOL 2010F, JEOL, Tokyo, Japan).

### 3.3. Preparation of Electrode

For working electrode, active materials, conductive reagent (Super P, MTI Co., Shenzhen, China), and binder (sodium alginate, CP grade, Sinopharm Chemical Reagent Co., Ltd.) were mixed and grounded with a mass ratio of 6:2:2. Then a certain amount of DI H_2_O was added in the mixture to produce slurry, which was cast on the Cu foil (MTI Co., thickness: approximately 10.5 μm). Then the electrode was dried in oven at 100 °C for 4 h and cut into discs with the diameter of 12 mm. The discs were then dried in a vacuum condition at 80 °C for 12 h and transferred into a glovebox (Superstar 1220, moisture and oxygen below 0.5 ppm, Mikrouna, Shanghai, China) filled with argon atmosphere for the fabrication of cells or pre-lithiated. The average mass loading of active material in each anode was around 0.86 mg cm^−2^.

For cathode, LiNi_0.6_Mn_0.2_Co_0.2_O_2_ (NMC622, Umicore Finland Oy, Kokkola, Finland) cathode was prepared as the following instruction: 80 wt% of NMC622, 10 wt% of Super P (conductive reagent) and 10 wt% of poly(1,1-difluoroethylene) (PVDF, binder, MTI Co.) were mixed in moderate N-Methyl pyrrolidone (NMP, 99%, J&K Scientific, Beijing, China,), then the obtained slurry was cast on the Al foil (GRINM Group, Beijing, China, thickness: 20 μm). After drying and cutting to the discs with a diameter of 12 mm, cathode electrodes were pressed at about 10 MPa for approximately 60 s. The average mass loading of NMC622 was 7.09 mg cm^−2^.

### 3.4. The Fabrication of Cells

CR2016 coin half cells were fabricated with working electrode, separator (made up of cellulose, NKK, city, Japan) and the reference electrode (lithium foil, Zhong Neng Co., Tianjin, China. Φ16 mm × 1.0). About 0.1 mL electrolyte containing 1 M LiPF_6_ in ethylene carbonate (EC)—diethyl carbonate (DEC) (1:1 vol%) containing 10% fluoroethylene carbonate (FEC) was added in coin cells. The electrolyte was produced by Nanjing Modges Energy Tech (Nanjing, China), and the water content is less than 10 ppm. Pre-lithiation process proposed by our reported work [19] was operated before full cells were fabricated. Full cells were fabricated by working electrode, separator and cathode. The N/P capacity ratio of full cells were 1.2 approximately. The same kind of separator and electrolyte were used in both full cells and half cells.

### 3.5. Electrochemical Test of Cells

Charging and discharging cycle was measured by CT2001A Land Batteries Testing System (Wuhan Land Electronic Co. Ltd., Wuhan, China). For cycling performance, half cells were discharged and charged at a small specific current of 100 mA g^−1^ for 5 cycles before a large specific current of 1000 or 2000 mA g^−1^. Full cells were charged and discharged at 0.1 C (18 mA g^−1^) based on the mass of cathode materials for several cycles.

Cyclic voltammetry (CV) and electrochemical impedance spectroscopy (EIS) test were operated on VSP-300 electrochemical workstation (Bio-Logic China Company, Shanghai, China). CR2016 coin half cells fabricated by the method mentioned in Section 3.4 were used to operate CV and EIS test. For the CV test, the voltage started at open circuit voltage (OCV) and reversed scan to 0.01 V and then scan to 2.0 V with a scan rate of 0.1 mV s^−1^. During the calculation of *D_Li^+^_*, the CV curves were obtained in different scan rate of 0.2, 1, 3, 5 and 10 mV s^−1^. For the EIS test, the frequency ranges from 1 MHz–1 mHz.

## 4. Conclusions

In conclusion, a cheap and convenient strategy to produce N-doped C-coating disproportionated SiO (d-SiO-NC) to be used as anode material for high-energy lithium ion batteries was reported. A relatively high reversible specific capacity (400 mA h g^−1^) and stable cycling performance could be delivered even at a high specific current of 2000 mA g^−1^. Based on the conclusion reported in literature [29], the addition of N could improve capacity and cycling retention further because some kinds of N, which were analyzed by XPS, could produce active sites in the carbon layer, contributing to the transport of lithium ions. Several ratios of d-SiO, pitch and melamine were investigated to get an overall good capability. CV and EIS tests were operated to investigate the mechanism of intercalation and extraction of active materials. Then, the pre-lithiation strategy was operated on d-SiO-NC anode electrode to improve the initial Coulombic efficiency. LiNi_0.6_Mn_0.2_Co_0.2_O_2_ (NMC 622) was used to assemble full cells to verify its practical application. What’s more, the low cost of pitch and melamine and the convenient producing process of this material contribute to its practical application in industry. The stability of cycling at a high current also makes it available on many kinds of instruments and situations.

## Figures and Tables

**Table 1:** The content of d-SiO, N and C in three kinds of prepared d-SiO-NC materials.

d-SiO:Melamine:Pitch	d-SiO Content	N Content	C Content
2:1:2	57.43%	4.73%	37.84%
2:1.5:3	46.88%	5.89%	47.13%
2:2:4	38.02%	6.89%	55.09%

**Table 2:** The values of ohmic, solid electrolyte film and charge transfer of d-SiO-C (2:4.5) and d-SiO-NC (2:1.5:3).

Sample	Conditions	*R_s_* (Ω)	*R_f_* (Ω)	*R_ct_* (Ω)
d-SiO-C (2:4.5)	Before cycle	2.269	285.3	~0
	After 5th cycles	2.097	20.03	49.39
	After 50th cycles	3.549	17.12	45.50
d-SiO-NC (2:1.5:3)	Before cycle	2.181	243.2	~0
	After 5th cycles	2.18	12.62	21.15
	After 50th cycles	2.40	12.81	13.12

**Table 3:** Warburg coefficient and lithium ion chemical diffusion coefficient calculated by EIS methods.

Cells	Conditions	*σ_W_* (Ω cm^2^ s^−0.5^)	*D_Li^+^_* (cm^2^ s^−1^)
d-SiO-C//Li	Before cycling	200.8	1.72 × 10^−16^
	After the 5th cycle	304.42	7.48 × 10^−17^
	After the 50th cycle	219.3	1.44 × 10^−16^
d-SiO-NC//Li	Before cycling	153.31	3.47 × 10^−16^
	After the 5th cycle	218.77	1.70 × 10^−16^
	After the 50th cycle	186.46	2.35 × 10^−16^

## Data Availability

Data is contained within the article.

## Sample Availability

Samples of the compounds d-SiO-C, d-SiO-NC and SiO-NC are available from the authors.
